# Impact of Alkali Metals on CeO_2_-WO_3_/TiO_2_ Catalysts for NH_3_-Selective Catalytic Reduction and Lifetime Prediction of Catalysts

**DOI:** 10.3390/molecules29235570

**Published:** 2024-11-25

**Authors:** Mutao Xu, Yuhang Deng, Xingxiu Gao, Qijie Jin, Wei Yan, Liguo Chen, Jian Yang, Jing Song, Changcheng Zhou, Haitao Xu

**Affiliations:** 1 School of Environmental Science and Engineering, Nanjing Tech University, Nanjing 210009, China; xmtxmt2019@163.com (M.X.); qijiejin@njtech.edu.cn (Q.J.);; 2 College of Materials Science and Engineering, Nanjing Tech University, Nanjing 210009, China; 3 Nanjing Gekof Institute of Environmental Protection Technology & Equipment Co., Nanjing 210031, China

**Keywords:** NH_3_-SCR reaction, Ce-based catalysts, alkaline metal poisoning, lifetime prediction

## Abstract

Ce-based catalysts have been widely used in the removal of nitrogen oxides from industrial flue gas because of their good catalytic performance and environmental friendliness. However, the mechanism of alkali metal poisoning in Ce-based catalysts remains to be further studied. This work involves the preparation of the K/Na-poisoned CeWTi catalyst via the impregnation method for assessing its performance in NO removal. Experiments show that both K and Na exhibit detrimental effects on the CeWTi catalyst, and the loading of alkali metal reduces the specific surface area and pore volume of the catalyst. Furthermore, the presence of alkaline metals results in a notable decline in the CeWTi acid concentration, particularly in Lewis acid sites. Concurrently, the levels of Ce^3+^, oxygen vacancies, and reducing agents on the catalyst surface decrease, leading to diminished reduction capability and eventual catalyst deactivation. The application of a BP neural network for catalyst activity prediction yielded an average relative error of approximately 0.73%, indicating enhanced accuracy in prediction outcomes. This work explored the cause of alkali metal poisoning of the CeWTi catalyst and provided an effective prediction method for the lifetime of CeWTi catalyst, which provided theoretical guidance for the engineering application of Ce-based catalysts.

## 1. Introduction

Nitrogen oxide (NO_x_) stands out as a prominent atmospheric pollutant, with its emissions triggering a myriad of environmental issues, including the formation of photochemical smog and acid rain [1,2]. These repercussions have been documented to inflict significant damage upon both the ecological landscape and human society [3,4]. The combustion of fossil fuels in the thermal-power-generation industry stands as a significant contributor to the emission of NO_x_ [5,6]. Nevertheless, there has been a noticeable rise in the proportion of NO_x_ emissions originating from non-power sectors, like iron and steel, cement, and waste incineration in recent years [7,8]. This shift has garnered considerable interest among scholars.

Presently, the utilization of NH_3_ as a reducing agent in selective catalytic reduction processes for the removal of NO_x_ pollutants from industrial flue gases has emerged as a prominent and highly effective denitrification technology [9,10]. The standard SCR reaction is

4NO + 4NH_3_ + O_2_→4N_2_ + 6H_2_O (1)

which contains a stoichiometry with identical amounts of NO and NH_3_ [11,12]. At the heart of this technology lies the development and enhancement of superior denitrification catalysts to achieve optimal performance. In general, the strong acidity and excellent redox properties of the catalyst are the two crucial factors that determine the effect of NH_3_-SCR technology, which affects the adsorption/activation of NH_3_ and NO_x_, while optimizing the reaction pathway of N_2_ and H_2_O generation [13]. Vanadium-based catalysts stand as the predominant choice for denitrification, with the V_2_O_5_-WO_3_(MoO_3_)/TiO_2_ catalysts demonstrating efficacy in industrial applications and featuring good anti-sulfur properties [14,15,16,17]. However, the principal active component of vanadium-based catalysts (V_2_O_5_) is a notably toxic substance, leading to concerns over secondary pollution arising during utilization and the environmental implications stemming from the disposal of deactivated denitrification catalysts following prolonged operation [6,18]. The advancement of eco-friendly catalysts tailored for the denitrification of industrial flue gases has emerged as imperative.

Cerium-based catalysts, representative of rare earth-based catalysts, have demonstrated remarkable catalytic efficacy and environmental benignity in the NH_3_-SCR reaction [19,20,21]. It has obvious synergistic effects when used in combination with components, such as metals or metal oxides, and exhibits far more reactivity than expected [22,23]. On this basis, a Ce-W-O_x_/TiO_2_ catalyst prepared via the impregnation method has excellent catalytic performance due to its high dispersion of active components [24,25,26]. The industrial flue gas of waste incineration, ceramics, and other industries contains a large number of alkali metals, among which the presence of typical alkali metals (K and Na) will lead to the poisoning and deactivation of catalysts [27]. The mechanism of alkali metal poisoning is complex and multifaceted, such as deposition on the catalyst surface, blocking the catalyst pore. The interaction with the acidic part of the catalyst surface leads to the overall acidity of the catalyst [28,29]. Therefore, it is of great significance for improving the low-temperature performance and stability of the Ce-W-O_x_/TiO_2_ catalyst to systematically explore the change law of catalytic performance and physical and chemical properties of the Ce-W-O_x_/TiO_2_ catalyst under the condition of alkali metal (K and Na) poisoning.

In addition, accurately forecasting the service life of cerium-based catalysts and establishing the optimal replacement cycle is essential for cost-effective NO_x_ treatment in flue gas processes [30]. With the development of computer technology, artificial intelligence technology has non-linear modeling capabilities, which are not affected by the reasons for product inactivation and service environment [31,32]. Notably, the BP neural network technique, serving as a feed-forward network that forwards signals and retroactively corrects errors to optimize the weights and thresholds across layers for superior outcomes, is particularly adept at processing multifaceted and intricate data sets, such as those related to SCR denitrification catalyst malfunction.

Typical alkali metal K and Na-loaded Ce-W-O_x_/TiO_2_ denitrification catalysts were synthesized using an impregnation method. After assessing the catalyst’s efficiency in reducing NO through performance testing and analyzing its physical and chemical properties, a plausible explanation for the catalyst’s alkali metal poisoning was determined. Furthermore, a life prediction model for cerium-based catalysts was developed using BP neural network technology. This study offers novel insights into understanding alkali metal poisoning in typical Ce-W-O_x_/TiO_2_ catalysts and forecasting the practical application longevity of the catalysts.

## 2. Results and Discussion

### 2.1. Catalytic Performance

**Figure 1:**
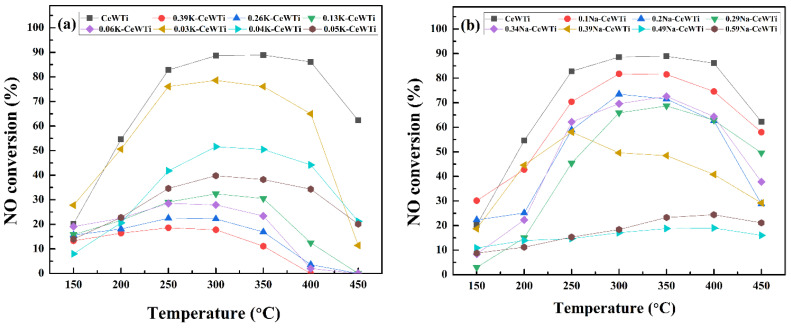
(**a**) NO conversion over K-CeWTi catalysts, (**b**) NO conversion of Na-CeWTi catalysts.

The catalytic performance of as-prepared catalysts for the NH_3_-SCR reaction was determined in Figure 1. Figure 1a shows that the NO conversion of the catalyst diminishes as the loading amount of K increases. When the molar ratio of K/Ce is below 0.13, the catalytic efficiency rises with temperature until reaching a peak at 300 °C, declining thereafter. Notably, a substantial decrease in efficiency is observed over 400 °C. Conversely, for molar ratios of K/Ce exceeding 0.13, the temperature at which peak efficiency occurs shifts to approximately 250 °C, with a significant drop in efficiency beyond 350 °C. Instances where the molar ratio of K/Ce reaches 0.06 or higher indicate that the optimal catalytic efficiency falls below 30%, signifying considerable deactivation. Particularly, the range from 0.03 to 0.04 in the K/Ce ratio, which exhibits the most pronounced decrease in efficiency, shows a decline of 34.4% at 300 °C. These findings underscore the susceptibility of the CeWTi catalyst to deactivation by even trace amounts of K, emphasizing its high sensitivity to minor changes in K loading levels.

Figure 1b illustrates a decline in the NO-conversion efficiency of the catalyst as the Na loading increased. It was noted that Na necessitated a higher loading quantity compared to K in order to induce an equivalent level of deactivation. Experimental observations indicated a marginal enhancement in catalytic efficiency with slight variations in Na loading at lower molar ratios of Na/Ce. This suggests that the CeWTi catalyst exhibited a degree of insensitivity to minor alterations in Na loading. When the molar ratio of Na/Ce was below 0.2, catalytic efficiency rose with an increasing temperature, reaching its peak at 300 °C, and decreased beyond 300 °C, with a notable decline observed post 400 °C. Upon surpassing a Na/Ce ratio of 0.29, the temperature at which maximum catalytic efficiency was achieved shifted towards higher values, around 350 °C, and further increased beyond 0.49. With a Na/Ce ratio exceeding 0.49, the optimal catalytic efficiency of the catalyst dropped below 30%, indicating significant deactivation. Hence, the impact of K on the CeWTi catalyst was evidently more pronounced than that of Na.

The K-CeWTi and Na-CeWTi catalyst was utilized as a case study to evaluate NO conversion at 300 °C. The activity of the catalyst at 300 °C was determined for varying levels of potassium loading using the designated catalyst activity calculation formula (Equations (2)–(4)). In these equations, *K* represents catalyst activity, *V* represents gas flow, A represents total surface area, $A_{v}$ represents surface velocity, $M_{R}$ represents the molar ratio of ammonia to nitrogen, η represents catalyst efficiency, $C_{slip,NH_{3}}$ represents ammonia escape concentration, and $C_{{NO}_{x},in}$ represents the inlet NO_x_ concentration to the SCR reactor.

(2) $$K=\left\{\begin{array}{c} 0.5\times A_{v}\times \ln\frac{M_{R}}{\left(M_{R}-\eta \right)\left(1-\eta \right)},\;M_{R}<1\\ -A_{v}\times ln\left(1-\eta \right),\;M_{R}\geq 1 \end{array}\right.$$

(3) $$A_{v}=\frac{V}{A}$$

(4) $$M_{R}=\frac{46}{17}\cdot \frac{C_{slip,NH_{3}}}{C_{{NO}_{x},in}}+\eta$$

**Figure 2:**
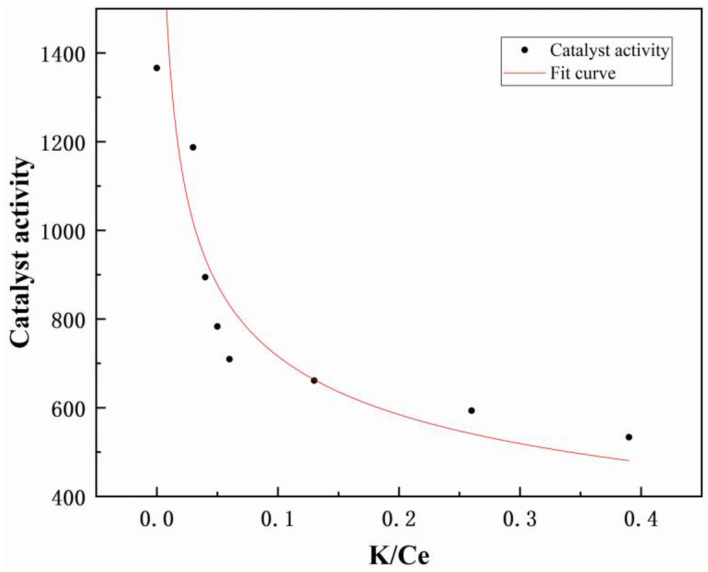
Fitting deactivation curve of K-poisoned CeWTi catalysts.

The derived catalyst activity data were analyzed using an allometric model. The deactivation curve, illustrated in Figure 2 and Figure S1, demonstrated the correlation between the loading of alkaline metal and the activity of the catalyst. Previous studies suggest that this curve signifies the selective nature of alkaline metal poisoning based on the CeWTi catalyst, wherein the most active sites within the catalyst are preferentially affected, leading to a swift deterioration in catalyst performance [33].

### 2.2. Morphologic, Structural, and Compositional Information

**Figure 3:**
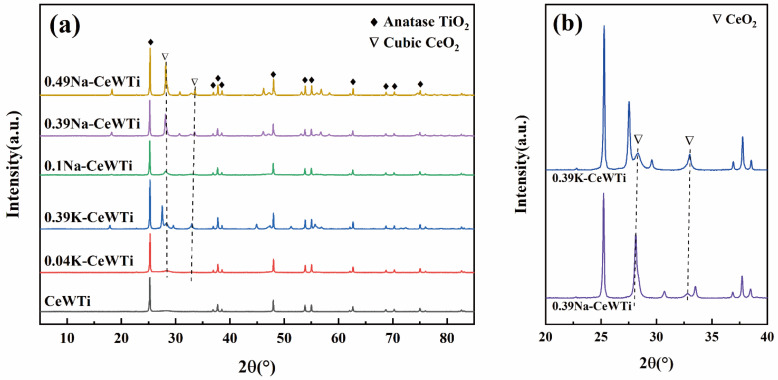
(**a**) XRD patterns of as-prepared catalysts, and (**b**) comparison of XRD patterns of 0.39K-CeWTi and 0.39Na-CeWTi at 20~40°.

In order to study the effect of different alkali metal loads on the phase composition of the CeWTi catalyst, the prepared catalyst samples were analyzed via X-ray diffraction, and the results are shown in Figure 3. The XRD patterns of the six samples unequivocally exhibited distinct anatase-type TiO_2_ diffraction peaks (ICDD-PDF 21-1272) while lacking any rutile-type TiO_2_ diffraction peaks [34,35]. This observation suggests that the high-temperature calcination process employed in the catalyst preparation did not induce a phase transformation from anatase to rutile in the TiO_2_ structure. Notably, in the CeWTi catalyst, the absence of discernible diffraction peaks corresponding to CeO_2_ and WO_3_ may be ascribed to the low crystallinity resulting from the limited presence of active components within the catalyst. It was highly dispersed on the surface of the TiO_2_, forming a single-layer distribution state. Moreover, with an increase in the loading of the alkali metal on the catalyst, the emergence of a CeO_2_ diffraction peak (ICDD-PDF 34-0394) was noted, implying that the addition of the alkali metal could promote catalyst sintering and the formation of microcrystalline CeO_2_, thereby diminishing the dispersion of the active component. According to the comparison of the local spectra of Figure 3b, the diffraction peak of CeO_2_ in the CeWTi catalyst loaded with Na is stronger, indicating that the crystal structure of the corresponding 0.39 Na-CeWTi catalyst is more intact and the crystal surface growth is more complete.

The specific surface area serves as an indicator of the extent of the interaction between the reaction gas molecules and the surface of the catalyst. Table 1 illustrates the surface area, pore volume, and average pore size of pre-poisoned catalysts. It is evident from the data that the specific surface area of the catalyst was notably influenced by the presence of K and Na, with the K-loaded catalyst exhibiting a more pronounced decrease. Notably, at a molar ratio of the alkaline metal to Ce of 0.39, the specific surface area decreased by 9.6 m^2^·g^−1^ for the K-loaded catalyst and by 10.6 m^2^·g^−1^ for the Na-loaded catalyst, corresponding to a reduction of 58.4%. The decline in pore volume mirrored that of the specific surface area, both diminishing as the alkaline metal content increased. Interestingly, the K-loaded catalyst experienced a lesser reduction in the pore volume compared to the Na-loaded catalyst, suggesting that Na had a more detrimental effect on the pore structure of the catalyst in comparison to K. Except for the significant change in the average pore size of the 0.1Na-CeWTi sample, the average pore size of other alkaline metal-loaded samples compared to fresh catalysts had little difference and no obvious variation pattern, which suggests that alkali metals exhibit minimal impact on the mean pore dimensions of the catalysts. In other words, the effect of alkali metals on the structure of the CeWTi catalyst is mainly reflected by the significant reduction in the specific surface area and pore volume of the catalyst. Previous studies have shown that alkali metal poisoning of denitrification catalyst can be divided into physical poisoning and chemical poisoning. For the physical poisoning of the catalyst, alkali metal will destroy the frame structure of the catalyst and reduce the specific surface area of the catalyst. In addition, the deposition of the alkali metal on the surface of catalyst will block the pore structure and seriously affect the diffusion of reactants [36,37]. Obviously, the larger the specific surface area, the more thorough the contact between the gas and the catalyst, the more advantageous the catalytic efficiency of the catalyst, which also explains the serious decline in the denitrification activity of the CeWTi catalyst with alkali metal poisoning.

**Figure 4:**
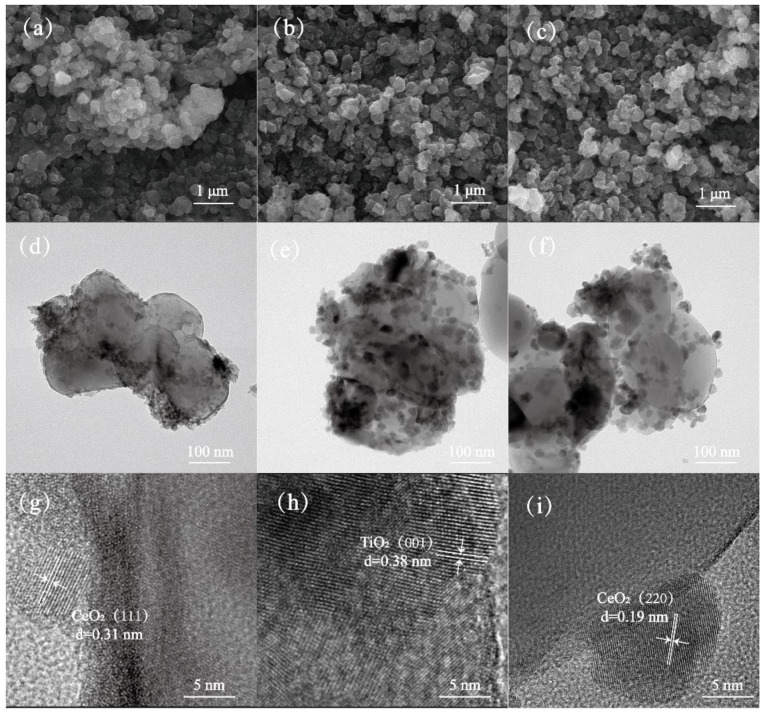
SEM image of as-prepared catalysts, (**a**) CeWTi, (**b**) 0.39K-CeWTi, (**c**) 0.39Na-CeWTi; TEM image of as-prepared catalysts, (**d**,**g**) CeWTi, (**e**,**h**) 0.39K-CeWTi, (**f**,**i**) 0.39Na-CeWTi.

The SEM and TEM image of as-prepared catalysts is shown in Figure 4. The results reveal that both the CeWTi catalyst and K/Na-CeWTi catalyst exhibit a common spherical particle morphology, with particle sizes falling within the range of 0.1 to 0.4 μm. This observation suggests that the impregnation method employed in the catalyst preparation led to a high degree of dispersion and uniform distribution of its components. As shown in the Figure 4g–i, the lattice fringes with a d value of 0.31 nm can be attributed to the (111) crystal plane of CeO_2_, the lattice fringes with a d value of 0.19 nm can be attributed to the (220) crystal plane of CeO_2_, and the lattice fringes with a d value of 0.38 nm are attributed to the (001) crystal plane of TiO_2_, respectively [38,39,40]. The main phase composition of the CeWTi catalyst in the XRD results was also verified. In addition, as shown in Figure 4e,f, the loaded components on the carrier were evenly distributed before the catalyst poisoning, and after the poisoning, the surface of the carrier was agglomerated and the particle size increased, which was also one of the reasons for the decrease in catalytic activity after the catalyst was poisoned by the alkali metal.

### 2.3. Surface Acid Analysis

**Figure 5:**
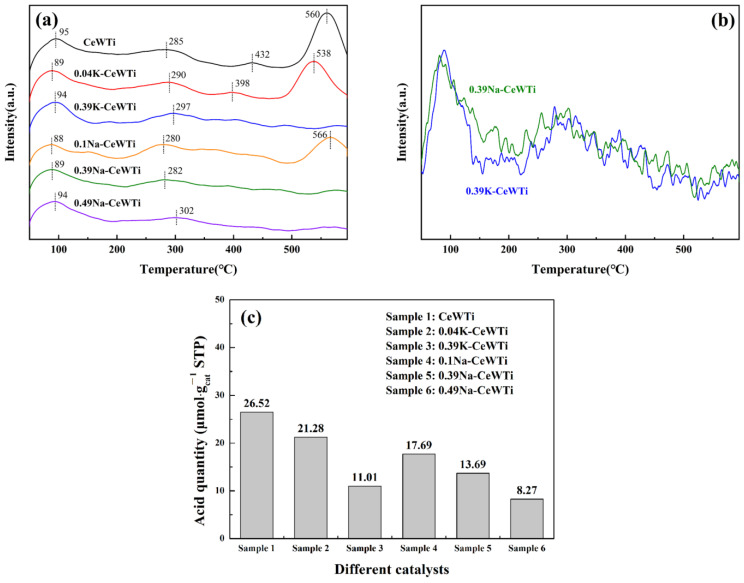
(**a**) NH_3_-TPD patterns of the as-prepared catalysts, (**b**) comparison of NH_3_-TPD patterns of 0.39K-CeWTi and 0.39Na-CeWTi, and (**c**) acid quantity of as-prepared catalysts.

The surface acidity of the catalyst is an important factor in SCR reactions. The acidic sites would affect the adsorption and activation of NH_3_ on the catalyst [41]. The quantity and strength of acid sites on the catalyst surface could be determined based on the desorption peak temperature and peak area of NH_3_-TPD. NH_3_-TPD tests were conducted on the six samples mentioned above to analyze the effect of alkaline metals on the acidity of the catalyst surface, and the test results are shown in Figure 5. All samples exhibited a low-temperature peek of NH_3_ desorption within 50~200 °C, which could be assigned to physisorbed NH_3_. With the increase in alkaline metal loading, there was no significant change in the desorption peak in this region. The rest of desorption region could be divided into three regions in the temperature ranges 200~350, 350~500, and 500~600 °C, assigned to NH_3_ desorbed from weak acid sites, medium-strong acid sites, and strong acid sites, respectively. The desorption peak at 200~350 °C belonged to the Brønsted acidic sites of the cerium species on the catalyst surface, while the desorption peak at 350~600 °C belonged to the stronger Lewis acidic sites [42]. Lewis acid sites mainly depended on the content of CeO_2_ [42]. Alkali metal will affect the redox performance of the catalyst, occupy or neutralize the Brønsted acid sites, weaken the Lewis acid sites, and reduce the surface acidity of the catalyst, thereby reducing the adsorption and activation of NH_3_ [43,44,45,46]. As shown in Figure 5, the fresh catalyst had the highest total acid amounts. When the loading of K/Na increased, the total acid amounts on the catalyst surface showed a downward trend, resulting in a decrease in the adsorption of ammonia gas, thereby reducing the denitrification activity of the catalyst. When the K/Ce ratio was 0.39, it could be found that the desorption peak area at 500~600 °C of the CeWTi catalyst loaded with K decreased more significantly. The attenuation of the number of acid sites at 500~600 °C indicated that the loading of alkaline metals would lead to a decrease in the number of metal ions on the catalyst surface that could accept isolated electrons, resulting in a reduction in the adsorption of reaction gas molecules.

### 2.4. Redox Performance

**Figure 6:**
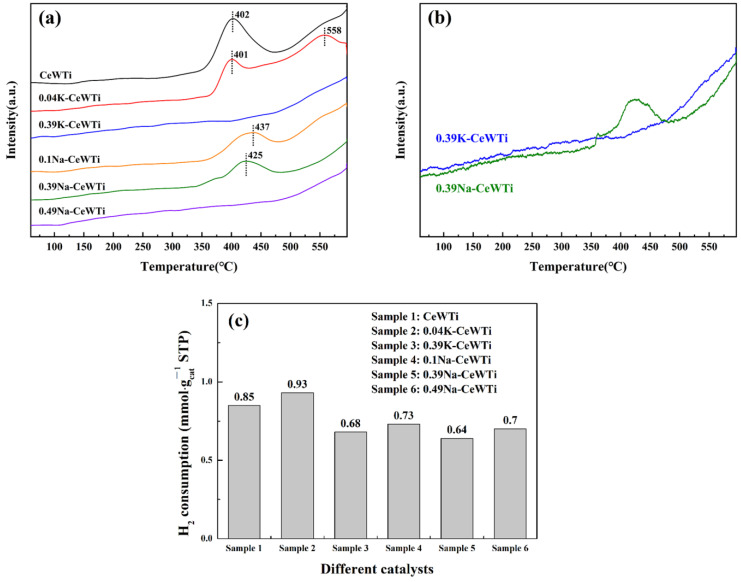
(**a**) H_2_-TPR patterns of the as-prepared catalysts, (**b**) comparison of H_2_-TPR patterns of 0.39K-CeWTi and 0.39Na-CeWTi, and (**c**) H_2_ consumption of as-prepared catalysts.

The reducibility of cerium species on the catalyst surface is another important factor in the SCR oxidation–reduction cycle reaction. The oxidation–reduction performance of the catalyst affected the catalytic reaction activity in the low-temperature region [47]. H_2_-TPR tests were conducted on the six samples mentioned above, and the results are shown in Figure 6. As shown in Figure 6, the catalyst showed a significant reduction peak around 401 °C, which is attributed to the reduction of adsorbed oxygen and lattice oxygen on the surface of the CeO_2_/WO_x_ solid solution [48,49,50]. This phenomenon is associated with the high dispersibility of the active metal oxide component, evident by the largest peak area, which signifies the highest total H_2_ consumption [26]. Consequently, it can be inferred that the fresh catalyst surface harbors more reducing species. Upon loading the catalyst with alkali metals, a gradual decline in the peak area was observed with increasing loading levels. Notably, when K/Ce ratio reached 0.39 and Na/Ce ratio reached 0.49, the reduction peak essentially vanished. The appearance of a broad peak around 558 °C may be ascribed to the reduction of surface/sub-surface oxygen of the crystalline CeO_2_ [51,52,53]. Following the loading of alkali metals, the reduction peak in this region became more prominent, indirectly suggesting that alkali metals could potentially disrupt the interaction between CeO_2_ and WO_x_ species. The shift in the reduction peak temperature towards higher temperatures observed in the Na-loaded catalyst suggests a decrease in the activity of Ce species on the poisoned catalyst’s surface. An analysis of XRD results indicates a potential reduction in the dispersion of CeO_2_ due to the presence of alkaline metals, leading to the formation of microcrystals. Moreover, BET results reveal a slightly greater decrease in the specific surface area for the Na-loaded catalyst compared to the K-loaded catalyst, consequently impacting its reduction capability.

### 2.5. Analysis of Electronic Properties

**Figure 7:**
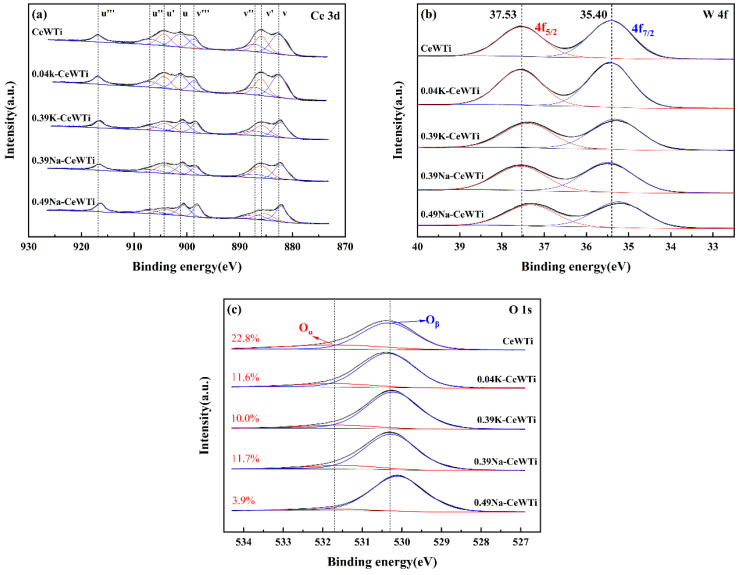
XPS spectra of the prepared catalysts, (**a**) Ce 3*d*, (**b**) W 4*f*, and (**c**) O 1*s*.

Alkaline metals could affect the redox performance of catalysts by affecting the valence state of the active component cerium species on the catalyst surface. Figure 7a shows the XPS spectra of Ce 3*d* for the prepared catalysts. Each spectrum had eight characteristic peaks, with peaks at u′ and v′ attributed to Ce^3+^ and peaks at u, u″, u‴, v, v″, and v‴ attributed to Ce^4+^ [52,54]. The results showed that the pattern of XPS performance was consistent with the activity test. When the molar ratio of the alkaline metal to Ce reached 0.39, the peak area attributed to Ce^3+^ exhibited a noticeable decrease, corresponding to a significant decrease in the catalytic performance of the denitrification catalyst under the alkaline metal loading in the activity test. The presence of Ce^3+^ played an important role in the Ce^3+^/Ce^4+^ redox cycle. When lattice oxygen ions left the CeO_2_ surface, oxygen vacancies would form, and the appearance of oxygen vacancies was accompanied by the production of active Ce^3+^, resulting in a state of valence imbalance, which enhanced the amount of adsorbates on the catalyst surface [55,56]. The decrease in the Ce^3+^ of alkaline metal-loaded catalysts provided evidence for the decrease in catalyst performance.

Figure 7b shows the XPS spectra of W 4*f* for prepared catalysts, the peaks of W 4f_5/2_ and W 4f_7/2_ at about 37.3~37.5 eV and 35.2~35.4 eV, respectively, attributed to W^6+^ species [57]. According to the results of other researchers, W^6+^ in WO_x_ was easily reduced to W^5+^, and the standard reduction potential from W^6+^ to W^5+^ was only −0.03 eV [58]. However, no reduced W^6+^ species were detected, indicating that WO_x_ was the electron donor group in the catalyst. The binding energies of W 4f in the alkaline-metal-supported catalysts decreased, and under the same molar loading ratio, the binding energies of W 4f in the KCl-supported catalyst decayed more, indicating a higher electron cloud density of W species on the catalyst, representing a decrease in the degree of electron transfer from WO_x_ to CeO_2_ in the catalyst.

Figure 7c shows the XPS spectra of O 1*s* for the prepared catalysts, which exhibited single-leaf-like peaks with left-right asymmetry and could be fitted by two characteristic peaks. The characteristic peak near 531.7 eV belonged to chemisorbed oxygen O_α_, and the characteristic peak near 530.3 eV belonged to lattice oxygen O_β_. It can be seen that the content of lattice oxygen was much richer than chemisorbed oxygen, but chemisorbed oxygen played an important role in oxidation reactions and has higher activity than lattice oxygen, which could promote the conversion of NO to NO_2_ [52,59]. Due to the presence of Ce^3+^, it led to an imbalance in the surface charge of the catalyst and the formation of unsaturated chemical bonds, which helped chemisorbed oxygen adsorb to the catalyst surface, which was consistent with the XPS spectrum results of Ce 3*d* [60]. When the alkaline metal loading increased, the content of Ce^3+^ decreased significantly, resulting in a decrease in adsorbed oxygen on the surface.

### 2.6. Lifetime Prediction of Catalysts

Catalyst lifetime prediction is a powerful tool for catalyst design and selection, as well as for assessing potential production costs, thus reducing operational expenses. The backpropagation neural network can effectively handle catalyst lifespan issues with relative accuracy. The construction of BP neural networks is typically carried out using Matlab. The steps for designing a BP neural network structure are as follows:

(1) Select data with a clear causal relationship with SCR catalyst activity as the input variables for the network.
(2) Due to the different units of each input variable, the input variables with larger values have a greater impact on the network. Therefore, it is necessary to normalize the input variables by using the mapminmax function in Matlab to normalize the data to between 0 and 1. The calculation formula is $y=\left(y_{max}-y_{min}\right)\times \frac{x-x_{min}}{x_{max}-x_{min}}+y_{min}$. *y_max_* and *y_min_* represent the set parameters, which can be set to 1 and 0, respectively, *x* represents the data that need to be normalized, *x_max_* represents the maximum value in the normalized data, and *x_min_* represents the minimum value in the normalized data.
(3) The number of hidden layer neurons is determined according to the empirical formula $n=\sqrt{m+d}+v$, where *m* represents the number of input-layer neurons, *d* represents the number of output-layer neurons, and *v* represents a constant and 1 < *v* < 10. The final choice is the number of hidden-layer neurons that minimizes the prediction error.
(4) A portion of the data is used as training samples to train the network, and the remaining data are used as test samples. The input variables of the test samples are substituted into the trained BP neural network, and the SCR catalyst activity prediction results are compared with the true values to analyze the error.
(5) The trained BP neural network can be exported for catalyst life prediction.

Based on the real-time flue gas parameter data of the SCR denitrification system, the key indicators affecting the activity of the catalyst were screened using correlation analysis. Combined with the obtained key indicators, the changing trend in the catalyst activity was predicted using the BP backpropagation neural network algorithm. Finally, the remaining life of the catalyst was determined based on the prediction results, providing a theoretical basis for the maintenance and replacement of the catalyst.

The BP neural network predicted the catalyst activity based on the flue gas parameters provided by the DCS system of the coal-fired power plant. The output value of the catalyst activity was compared with the expected value, and the weight value was adjusted to minimize the error. Through the Spearman correlation analysis process, the network input parameters were determined to be the operation time, unit load, SCR reactor ammonia addition, SCR reactor denitrification efficiency, air preheater pressure loss, and SCR reactor inlet flue gas flow. A three-layer BP neural network was used for prediction, with six neurons in the input layer, eight neurons in the hidden layer, and one neuron in the output layer. After data preprocessing, the data set was divided into two groups according to a ratio of 4:1, with the former serving as the training group and the latter as the testing group. In this project, six key indicators affecting the catalyst activity would be determined as the input parameters of the BP neural network, and the catalyst activity would be the output parameter of the network to predict the activity of the catalyst. Table 2 shows the input neural network parameters.

**Figure 8:**
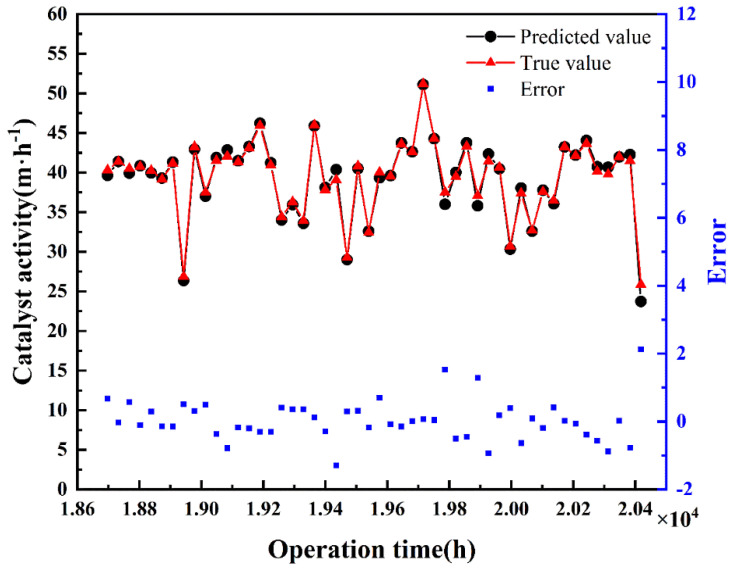
Comparison of predicted values and true values of BP neural network test.

To examine the effectiveness of the model, one-year data were selected for verification, with data collected every half hour. The corresponding catalyst operating time range was 11,689~20,449 h. The one-year operating data basically covered the main operating conditions of the unit. After data validity processing, the catalyst activity was predicted. As shown in Figure 8, catalyst activity under operating conditions fluctuated to a certain extent within the long-term operating time range, and the data-prediction curve showed a trend similar to the actual activity curve. Further analysis of the error between the predicted value and the true value shows that the average relative error of the test sample is at least 0.73% based on the sample data of the study. Therefore, the prediction model based on the BP neural network structure can be used to predict the operating performance of the CeWTi catalyst, and give more accurate prediction results.

## 3. Experimental

### 3.1. Catalyst Preparation

Ce-W-O_x_/TiO_2_ and alkali-metal-poisoned catalysts were prepared via the impregnation method [61]. The molar ratio of each element in the Ce-W-O_x_/TiO_2_ catalyst was Ce:W:Ti = 1:1:10. First, equal masses of citric acid monohydrate and ammonium metatungstate were dissolved in the water. Then, an appropriate amount of Ce(NO_3_)_3_·6H_2_O was added under the condition of continuous stirring. In order to study the toxic effect of alkali metals (K, Na) on the catalyst, they were loaded on the catalyst at the preparation stage. A certain amount of KCl or NaCl was added with Ce(NO_3_)_3_·6H_2_O in this step. Next, the appropriate amount of TiO_2_ was added and stirred for 2 h. Finally, the solid was dried in a blast drying oven at 100 °C for 5 h and calcined at 500 °C for 5 h. The alkali metal-supported catalysts were named X K/Na-CeWTi (X stands for the molar ratio of K/Na to Ce). Using inductively coupled plasma optical emission spectrometry (ICP-OES) on an Avio 500 from PerkinElmer, Inc. (Cambridge, MA, USA), the actual elemental compositions were analyzed, and quantitative data are listed in Table S1.

### 3.2. Catalytic Activity Measurement

Details of the activity assessment and characterization are presented in Supplementary Material (Text S1. Experimental section).

## 4. Conclusions

In general, we prepared K- and Na-poisoned CeWTi catalysts via the impregnation method to explore the effect of alkali metal poisoning on the NH_3_-SCR denitrification performance of Ce-based catalysts. Both K and Na can cause a significant decrease in the NH_3_-SCR activity of CeWTi catalysts. The NO conversion rates of different catalysts were evaluated at 300 °C, resulting in 88.6% for the CeWTi catalyst, 49.6% for 0.39Na-CeWTi, and merely 17.8% for 0.39K-CeWTi. Following alkali metal poisoning, the denitrification efficiency of the CeWTi catalyst notably diminished. Particularly, the detrimental impact of K on the CeWTi catalyst surpassed that of Na, indicating a more pronounced toxicity of K towards the catalyst. Concretely, the loading of alkali metal reduces the specific surface area and pore volume of the catalyst. Furthermore, the presence of alkaline metals results in a notable decline in the CeWTi acid concentration, particularly in Lewis acid sites. Concurrently, the levels of Ce^3+^, oxygen vacancies, and reducing agents on the catalyst surface decrease, leading to diminished reduction capability and eventual catalyst deactivation. The application of a BP neural network for catalyst activity prediction yielded an average relative error of approximately 0.73%, indicating enhanced accuracy in prediction outcomes. This work explored the cause of alkali metal poisoning of the CeWTi catalyst and provided an effective prediction method for the lifetime of the CeWTi catalyst, which provides theoretical guidance for the engineering application of Ce-based catalysts.

## Figures and Tables

**Table 1:** BET surface area, pore volume, and average pore size of as-prepared catalysts.

Samples	Surface Area (m^2^·g^−1^)	Pore Volume (cm^3^·g^−1^)	Average Pore Size (nm)
CeWTi	18.2	0.06	13.8
0.04K-CeWTi	14.4	0.06	15.8
0.39K-CeWTi	8.6	0.04	14.0
0.1Na-CeWTi	11.7	0.08	23.3
0.39Na-CeWTi	7.6	0.03	10.9
0.49Na-CeWTi	6.7	0.03	12.3

**Table 2:** Parameter name and setting of BP neural network.

Parameter Name	Parameter Setting
Input layer activation function	tansig
Implied layer excitation function	tansig
Output layer excitation function	purelin
Network training function	trainlm
Number of learning iterations	1000
Network learning accuracy	0.00001

## Data Availability

The authors declare that all data supporting the findings of this study are available within the article.

## Supplementary Materials

The following supporting information can be downloaded at: https://www.mdpi.com/article/10.3390/molecules29235570/s1, Table S1: Elemental contents of as-prepared catalysts. Figure S1: Fitting deactivation curve of Na-poisoned CeWTi catalysts.
