# Supplementary material for: Impact of Alkali Metals on CeO2-WO3/TiO2 Catalysts for NH3-Selective Catalytic Reduction and Lifetime Prediction of Catalysts
Source: Molecules. 2024 Nov 25;29(23):5570. doi: 10.3390/molecules29235570 (PMC11643063; doi:10.3390/molecules29235570)
Supplement: Supplementary file 1 [file molecules-29-05570-s001.zip › molecules-3301442-supplementary.pdf]

Supplementary Material for

Impact of alkali metals on CeO_2-WO_3/TiO_2 catalysts for NH_3-SCR and lifetime prediction of catalyst

Mutao Xu[a,b†], Yuhang Deng[a†], XingXiu Gao[a], Qijie Jin[a,c]*, Wei Yan[b], LiGuo Chen[a], Jian Yang[b]*, Jing Song[c], Changcheng Zhou[c], Haitao Xu[a,c]*

a. School of Environmental Science and Engineering, Nanjing Tech University, Nanjing 210009, PR China

b. College of Materials Science and Engineering, Nanjing Tech University, Nanjing 210009, PR China

c. Nanjing Gekof Institute of Environmental Protection Technology & Equipment Co., Nanjing 210031, PR China

†These authors contributed equally to this work.

*Corresponding authors: Haitao Xu, Qijie Jin, Jian Yang

E-mail address: htxu@njtech.edu.cn, qijiejin@njtech.edu.cn, yangjian1976@163.com

Text S1. Experimental section

1. Chemicals and Materials

All chemicals were purchased from commercial sources and used without further treatment. $(NH_4)_6H_2W_{12}O_{40}\cdot xH_2O$, TiO_2(anatase), $C_6H_8O_7\cdot H_2O$, $Ce(NO_3)_3\cdot 6H_2O$, KCl, NaCl were purchased from Shanghai Macklin Biochemical Technology Co., LTD. The water used is deionized from laboratory preparation.

2. Supporting table

Table S1. Elemental contents of as-prepared catalysts

Samples	Elemental concentration (wt.%)	
	K	Na
CeWTi	/	/
0.04K-CeWTi	0.13	/
0.39K-CeWTi	1.21	/
0.1Na-CeWTi	/	0.19
0.39Na-CeWTi	/	0.72
0.49Na-CeWTi	/	0.93

3. Catalytic activity measurement

The catalyst (1.0 mL) was added to a fixed-bed quartz reactor to investigate the catalytic performance. The 400 $mL\cdot min^{-1}$ gas flow rate corresponded to a GHSV of 24000 h^{-1}. The reactant gas was composed of 500 ppm NO, 500 ppm NH_3, 5 vol.% O_2 and balance N_2. The reaction temperature ranges from 100 °C to 500 °C, and the gradient is 50 $°C\cdot 15\ min^{-1}$. After the reaction reached a steady state (30 min), the catalytic activity at the corresponding temperature point was recorded. The NO concentration at the inlet and outlet of the reactor was detected by a flue gas analyzer (KM950, Kane International). The catalytic activity of NO was calculated by equation (S1).

$$\eta_{NO} = \frac{NO_{in} - NO_{out}}{NO_{in}} \times 100\% \qquad \text{Eq. (S1)}$$

Among them, $[NO]_{in}$ represented the NO concentration at inlet of the reactor, $[NO]_{out}$ represented the NO concentration at outlet of the reactor.

4. Details of characterization instrument and measurement procedures

X-ray diffraction (XRD) patterns were obtained from an X-ray diffractometer (Smartlab TM 3 kW, Rigaku, Japan). The scan speed was 10 °·min^{-1} and the 2θ scans covered 10~85 °.

The specific surface area and average pore diameter (BET method) of the samples were measured by N_2 adsorption/desorption isotherms at -196 °C using a surface-area analyzer (Micromeritics, 2020M V3.00H). All of the samples were degassed at 350 °C under vacuum for 3 h prior to the adsorption experiments.

The microstructural natures of the catalysts have been investigated using a transmission electron microscopy (JEOL, JEM-2010UHR).

X-ray photoelectron spectroscopy (XPS) patterns were acquired by an AXIS ULTRA DLD instrument (Al-Kα radiation, 1486.6 eV), and the vacuum degree was maintained at 10^{-7} Pa. The samples were dried at 100 °C for 24 h to remove moisture and then were tested without surface treatment. The curve fitting was performed by using XPSPEAK 4.1 with a Shirley-type background.

The temperature programmed desorption of ammonia (NH_3-TPD) was conducted on the CHEMBET-3000 (Quantachrome) to obtain the surface acid properties. All the catalysts were preheated at 400 °C under a helium stream for 1 h, and then cooled to 50 °C for the ammonia adsorption. Afterwards, ammonia was desorbed from 50 °C to

800 °C at a heating rate of 10 °C·min^{-1}.

The Semiautomatic Micromeritics TPD/TPR 2900 instrument was used for the temperature programmed reduction of hydrogen (H_2-TPR). All the catalyst carriers were preheated to 400 °C under an argon stream for 1 h, and cooled to 50 °C. Then 5% H_2/Ar flow was switched, and the temperature increased from 50 °C to 800 °C at a 10 °C·min^{-1} heating rate. The data were collected throughout the whole temperature range.

5. Supporting figure

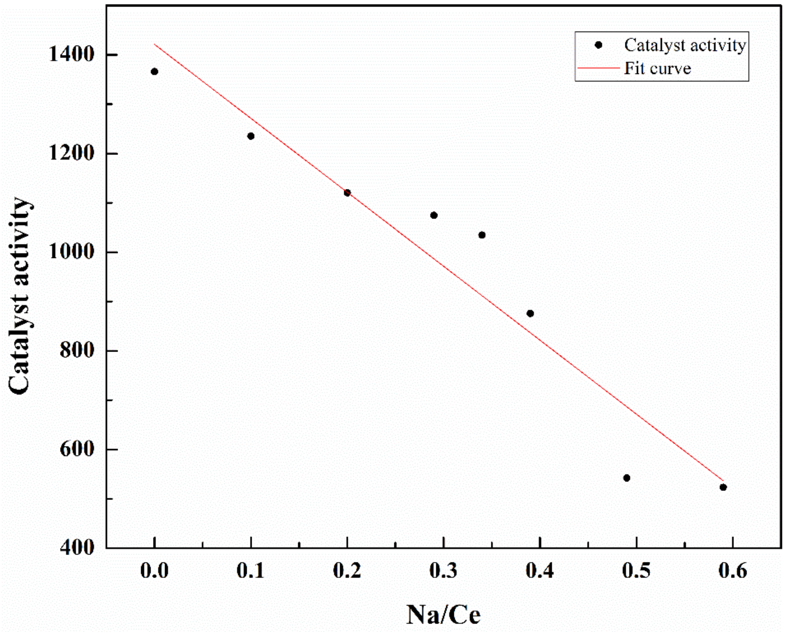

Fig. S1 Fitting deactivation curve of Na-poisoned CeWTi catalysts.
